# A Holistic Perspective: Exosomes Shuttle between Nerves and Immune Cells in the Tumor Microenvironment

**DOI:** 10.3390/jcm9113529

**Published:** 2020-10-31

**Authors:** Mihnea P. Dragomir, Vlad Moisoiu, Roxana Manaila, Barbara Pardini, Erik Knutsen, Simone Anfossi, Moran Amit, George A. Calin

**Affiliations:** 1 Department of Surgery, Fundeni Clinical Hospital, Carol Davila University of Medicine and Pharmacy, 022328 Bucharest, Romania; 2 Institute of Pathology, Charité University Hospital, 10117 Berlin, Germany; 3 Faculty of Physics, Babeş-Bolyai University, 400084 Cluj-Napoca, Romania; vlad.moisoiu@gmail.com; 4 Clinical Institute of Urology and Renal Transplantation, 400006 Cluj-Napoca, Romania; roxanamanaila@gmail.com; 5 Italian Institute for Genomic Medicine (IIGM), 10060 Candiolo, Italy; barbara.pardini@iigm.it; 6 Candiolo Cancer Institute, FPO-IRCCS, 10060 Candiolo, Italy; 7 Department of Medical Biology, Faculty of Health Sciences, UiT—The Arctic University of Norway, N-9037 Tromsø, Norway; erik.knutsen@uit.no; 8 Department of Translational Molecular Pathology, The University of Texas MD Anderson Cancer Center, Houston, TX 77030, USA; sanfossi@mdanderson.org; 9 Department of Head and Neck Surgery, The University of Texas MD Anderson Cancer Center, Houston, TX 77030, USA; MAmit@mdanderson.org; 10 The Center for RNA Interference and Non-Coding RNAs, The University of Texas MD Anderson Cancer Center, Houston, TX 77030, USA

**Keywords:** tumor microenvironment, immune system, neurotropic carcinoma, neurogenesis, exosomes, microRNAs

## Abstract

One of the limitations of cancer research has been the restricted focus on tumor cells and the omission of other non-malignant cells that are constitutive elements of this systemic disease. Current research is focused on the bidirectional communication between tumor cells and other components of the tumor microenvironment (TME), such as immune and endothelial cells, and nerves. A major success of this bidirectional approach has been the development of immunotherapy. Recently, a more complex landscape involving a multi-lateral communication between the non-malignant components of the TME started to emerge. A prime example is the interplay between immune and endothelial cells, which led to the approval of anti-vascular endothelial growth factor-therapy combined with immune checkpoint inhibitors and classical chemotherapy in non-small cell lung cancer. Hence, a paradigm shift approach is to characterize the crosstalk between different non-malignant components of the TME and understand their role in tumorigenesis. In this perspective, we discuss the interplay between nerves and immune cells within the TME. In particular, we focus on exosomes and microRNAs as a systemic, rapid and dynamic communication channel between tumor cells, nerves and immune cells contributing to cancer progression. Finally, we discuss how combinatorial therapies blocking this tumorigenic cross-talk could lead to improved outcomes for cancer patients.

## 1. Introduction

### 1.1. A New Perspective on the Tumor Microenvironment

Cancer is a devastating disease that has become the leading cause of death in developed countries [1], despite enormous efforts to decipher the key molecular events responsible for its onset and progression. All solid tumors are composed of cancer cells embedded between several types of non-malignant specialized cells which are essential to tumorigenesis and comprise the tumor microenvironment (TME) [2]. The TME is a unique environment emerging during tumor progression as a result of the interactions between tumor cells and the host. It is created, continuously modified and dominated by the tumor, which orchestrates molecular and cellular events taking place in surrounding tissues [3]. Apart from cancer cells, the TME is a complex mixture of endothelial cells (responsible for neo-angiogenesis), pericytes, immune cells (responsible for immune distraction and tumor promoting inflammation), fibroblasts, undifferentiated progenitor cells [2] and, according to more recent studies, nerves [4]. Current research focuses mainly on the bidirectional communication between tumor cells and the other components of the TME. An important success of this bidirectional approach was the development of immunotherapy, especially of the monoclonal antibodies against cytotoxic T-lymphocyte-associated protein 4 (CTLA-4) and programmed cell death protein 1 (PD1)/ programmed death-ligand 1 (PD-L1). Unfortunately, only a minority of cancer patients attain complete response to immunotherapy [5], thus novel therapeutic strategies must be developed. A growing body of evidence suggest that elements of the TME can directly influence each other, an example being the interplay between immune and endothelial cells. Innate immunity cells can alter vascular endothelial growth factor (VEGF) receptor expression [6,7]. This led to the association of anti-VEGF-A therapy with immune checkpoint inhibitors and classical chemotherapy in non-small cell lung cancer (NSCLC). Very interestingly, this combination led to prolonged overall survival (OS) of treatment-naive stage IV NSCLC patients [8]. It may be speculated that the three drugs do not act separately, but together: the cytotoxic activity of the chemotherapy combined with the vascular permeabilization induced by the anti-VEGF-A provoke an important release of tumor antigens, hence aiding the immune checkpoint therapy [6]. This example underscores a completely new approach to cancer treatment based on the disruption of the cross-talk between the different non-malignant components of the TME. Along similar lines, recent evidence suggests that there is an interplay between nerves and immune cells within the TME [9]. We consider that understanding this relationship could lead to new combinatorial therapies that might improve cancer outcomes. Of note, both immune cells and neurons are spread throughout the body and can communicate in areas distant from the tumor. Therefore, the communication mechanism must be systemic, and dynamic. 

Quail and Joyce reported many examples of heterotypic signaling within the elements of TME which involved classical paracrine signaling loops of cytokines or growth factors and their receptors [10]. Together with these key mechanisms of intercellular communication within the TME, extracellular vesicles (EVs) have emerged as an additional way of cell–cell communication [11]. In many types of solid tumor, cancer-derived exosomes from the primary tumor “prepare” the microenvironment to form a pro-tumorigenic niche, and direct bone marrow-derived progenitors to enhance and promote metastatic dissemination [12]. For example, exosomes derived from aggressive melanoma cells increased growth and metastasis of primary tumors, and programmed bone marrow-derived cells at the pre-metastatic site to acquire a pro-angiogenic phenotype. Interestingly, the mechanism was due to the transport via exosomes of the receptor tyrosine kinase MET Proto-Oncogene (MET) that, if inhibited, impaired pro-metastatic effects [13]. Hence, we allege that exosomes and their cargo might contribute to this complex interplay. 

### 1.2. Exosomes and microRNAs Represent a Systemic, and Dynamic Communication Channel between the Components of the Tumor Microenvironment

Traditionally, intercellular communication was thought to be mediated through direct cell–cell contact or via transfer of secreted chemical messenger or macromolecules such as hormones [14]. In the last two decades, a third mechanism for intercellular communication has emerged that involves intercellular transfer of EVs [15]. There are various types of secreted EVs that have distinct structural and biochemical properties depending on their intracellular site of origin; however, they are still poorly characterized and there is lack of a consensus in the nomenclature [16]. Exosomes, a specific subtype of EVs, play a significant role in intercellular communication by serving as a carrier for the transfer of membrane and cytosolic proteins, lipids, and RNA between cells. They arise intracellularly via inward budding of the cell membrane of the endosomes and are released upon exocytosis of multivesicular bodies [17,18]. Exosomes have a spherical structure, limited by a lipid bilayer, a 40–100 nm size in diameter, and a cup-shaped appearance by electron microscopy. Exosomes display specific protein markers such as tetraspanins (CD63, CD9), heat shock proteins (HSP70 and HSP90) as well as a wide range of other proteins which dictate the cell types they target [19]. 

Each cell type is able to turn on exosomes’ biogenesis depending on the physiological state and further control the sorting of exosomal cargo [20]. The secretion of exosomes can be spontaneous or induced depending on the cell type and environmental changes, such as hypoxia. T cells, mastocytes, and resting B cells secrete detectable levels of exosomes into the blood following the activation of specific cell surface receptors [21]. By contrast, most tumor cell lines and other immune cells, such as dendritic cells (DC) and macrophages, constitutively secrete exosomes in vitro [21]. In addition, tumor cells release exosomes at a greater rate than normal cells [21].

Exosomes and other EVs represent the way donor cells communicate with recipient cells and influence their gene expression [22]. Exosomes have antigen-dependent immune functions, as they carry antigenic material and peptide–major histocompatibility complex (MHC) but also antigen independent immune functions. RNA sequencing and proteome analyses of exosomes revealed that these vesiclescarry both proteins and RNA molecules, including messenger RNAs (mRNA), and non-coding RNAs (ncRNAs), such as microRNAs (miRNAs) and long non-coding RNAs (lncRNAs) [23,24,25], which are transferred to recipient cells [26]. As exosomes enter their target cells and release their cargo, they modulate cell functions and even prompt identity switching [26]. The first to describe the cell–cell communication mediated by RNAs included in exosomes was Valadi et al. in 2007: exosomes carried miRNAs and other RNAs from one cell to another and, when released in the target cell, were able to interact with the gene expression machinery to modify the gene expression profile of the recipient cell [27].

The role of miRNAs as mediators for intercellular communication with a hormone-like mechanism has also recently been described [23,28]. Tumor-derived exosomes containing miRNAs can in fact directly modify tumor cell invasiveness and motility through modification of the TME [29,30]. Interestingly, the ectopic expression of miR-409 in normal prostate fibroblasts conferred a cancer-associated stroma-like phenotype and the release of this miRNA via exosomes was able to promote tumorigenesis and epithelial-mesenchymal transition (EMT) through repression of Ras suppressor 1 (RUS1) and stromal antigen 2 (STAG2) [31]. 

Here, we focus on tumor-secreted exosomes and their miRNA cargo as an alternative communication channel between the tumor, immune, and nerve cells. Recent research has highlighted the impact of tumor-derived exosomes on the immunosuppressive TME and on tumor-associated nerve cells.

## 2. Communication between Nerve Cells and Cancer Cells

### 2.1. Nerves within the Tumor Microenvironment Can Either Promote or Inhibit Tumorigenesis

The autonomic nervous system is the part of the peripheral nervous system which maintains body homeostasis by relaying information between the central nervous system (CNS) and the periphery (sensors and effectors) [32]. The autonomic nervous system is further divided in the sympathetic (SNS) and parasympathetic nervous systems (PSNS). Both the SNS and PSNS have an afferent nerve fibers connected to special sensory organs (nociceptors, chemosensors, pH sensors) and an efferent component honing on effectors represented by smooth muscles, cardiac muscles and glands. The specific cellular architecture of the autonomous afferents consists of a series of two neurons connected within peripheric ganglions: a preganglionic neuron originating within the CNS, and a postganglionic neuron linked to effectors. The unique localization of postganglionic neurons outside the CNS but also outside the tissue they innervate have two important consequences: on the one hand, being outside the CNS, postganglionic neurons are in a position to adapt their transcription, translation and cytoskeletal dynamics to the TME they innervate independently of the CNS. Similarly, emerging evidence suggest that sensory neurons and the TME exert mutual influence on each other [33]. On the other hand, because of the location of postganglionic neuron bodies outside the malignant tissue, studies focusing on the malignant tissue itself fail to capture the molecular signals of postganglionic neurons, resulting in an underappreciation of their effect on the TME. SNS and PSNS exert their function through a series of specific transmitters and cognate receptors. A summary of the adrenergic and cholinergic receptor subtypes, effectors and ligands is presented in Table 1. 

In addition to being neurotransmitters, adrenaline and noradrenaline are also stress hormones released systemically by the adrenal medulla [34]. In fact, the adrenal medulla can be considered as a specialized sympathetic ganglion as it is innervated by preganglionic nerve fibers. Unlike sympathetic ganglia, the adrenal medulla lacks synapses and its secretions are released directly into the blood [35]. Thus, the role of autonomous nervous system (sympathetic and parasympathetic signaling) must be understood in the broader context of adaptation to stress, which involves both nerves and hormones.

Nerves and blood vessels often travel in tandem as neurovascular bundles and both play crucial roles in the development and maintenance of organs. Not unexpectedly, an increase in blood vessel and nerve density is a hallmark histological change in cancer. Conceptually, there is a distinction between cancer-induced growth of nerves and nerve-induced growth of cancer cells. On the one hand, cancer cells induce neuritogenesis (formation of neurits), which can further develop into axons (axonogenesis = sprouting of new nerve fibers) and, more rarely, neurogenesis (proliferation of neurons). On the other hand, nerves can stimulate tumor progression by forming a positive feedback loop within the TME [36]. The invasion of nerves by malignant cells is termed perineural invasion (PNI) [37]. PNI is a marker of unfavorable outcome and is associated with shorter overall survival (OS) in gastric, colon, rectal, prostate, esophageal, biliary tract, head and neck, and other cancers [4,38,39,40,41,42,43]. In addition to having a prognostic role, PNI was proposed as an indication for a more aggressive treatment approach, like radiotherapy, in head and neck cancers [44]. 

Early evidence concerning the nerve-tumor relationships emerged from tumor denervation studies suggesting that nerves within the TME can either promote or inhibit tumorigenesis depending on tumor type. Thus, surgical transection of the vagus nerve (which severs both parasympathetic and sensory axons in this mixed nerve) accelerates progression from pancreatic intraepithelial neoplasia to pancreatic ductal adenocarcinoma (PDAC) [45,46], while in gastric cancer, the surgical transection of the vagus nerve has anti-tumorigenic effects [47]. The difference stems from the fact that most gastric cancers are adenocarcinoma arising from the glandular epithelium that receive cholinergic afferents, while PDACs are derived from the ductal epithelium receiving a great deal of adrenergic innervation. In vivo studies in prostate cancer showed that both the sympathetic and parasympathetic nerves of the TME can promote tumorigenesis. The sympathetic nerve fibers regulate the initial growth phase of prostate cancer via the β 2 and β3 adrenergic receptors (AR) on stromal cells, while the parasympathetic fibers control invasion and metastasis via type 1 muscarinic acetylcholine receptor (M_1_-AChR) on stromal cells [48]. 

Dissecting the specific contribution of sympathetic and parasympathetic afferents as well as of sensory autonomous neurons to cancer onset and progression requires a variety of strategies involving genetic manipulation (i.e., transgenic mice), optogenetics, neurotoxins, pharmacological modulation, and surgical denervation [49]. Using chemical denervation of the sensory component of the vagus nerve, a clear anti-tumorigenic effect of sensory autonomous nerves in PDAC emerged [33,50,51]. The emerging landscape suggests that adrenergic and autonomous sensory neurons have a pro-tumorigenic effect in solid malignancies, while the effect of parasympathetic signaling is cancer-type dependent (Table 2). The situation differs, however, in the case of hematological malignancies, where adrenergic signaling has an anti-tumorigenic effect. 

An extensive body of evidence demonstrated that adrenergic signaling has pro-tumorigenic effects in lung cancer (reviewed in [52]). In NSCLC, Nilsson and colleagues described a mechanism of resistance to anti-epidermal growth factor receptor (EGFR) inhibition involving beta 2-adrenergic receptors (β_2_-AR) signaling [53]. The authors showed that adrenaline activates β_2_-AR on NSCLC cells, which cooperatively signals with mutant EGFR to induce IL-6 expression through the tumor suppressor, liver kinase B1 (LKB1). Similarly to adrenergic signaling, cholinergic signaling also has pro-tumorigenic effects in lung cancer (reviewed in [54]) and the density of both types of nerve fibers is associated with a worse outcome in lung adenocarcinoma [55]. Zhao et al. have shown that biochemical events downstream M_2_AChR activation include the activation of NF-κB p65, which drives migration and invasion and promotes EMT. 

In gastric cancer, both sympathetic and parasympathetic signaling was shown to have pro-tumorigenic effects. The pro-tumorigenic effects of adrenergic signaling is mediated through several signaling pathways stemming from β_2_-AR, including the extracellular signal-regulated kinases 1/2 (ERK1/2), c-Jun N-terminal kinases (JNKs) and mitogen-activated protein kinase (MAPK) pathways. The transcription factors mediating the pro-tumorigenic effect downstream of β_2_-AR include nuclear factor kappa-light-chain-enhancer of activated B cells (NF-κB), activator protein 1 (AP-1), cAMP response element-binding protein (CREB) and (signal transducer and activator of transcription 3) STAT3 [56] and while AMP-activated protein kinase (AMPK)-dependent autophagy was also shown to [57]. In addition, catecholamine-induced β_2_-AR activation mediates desensitization of gastric cancer cells to EGFR antibodies (transtuzumab) by upregulating Mucin 4 (MUC4) expression [58]. In regard to cholinergic signaling, Zhao et al. reported that the pro-tumorigenic effects of cholinergic signaling takes place via M3 receptor–mediated Wingless and Int-1 (Wnt) signaling in the stem cells [47] while Hayakawa et al. reported that acetylcholine stimulates tumor growth via its M_3_-AChR, activating yes-associated protein (YAP), a modulator of Wnt/β-catenin signaling [59]. 

The pro-tumorigenic effects of adrenergic signaling in colorectal cancer is dependent on EGFR- protein kinase B (PKB/Akt)/ERK1/2 signaling [60], while Han et al. unveiled a norepinephrine-CREB1-miR-373 axis that promotes the progression of colon cancer by downregulating tissue inhibitor of metalloproteinases (TIMP2) and adenomatous polyposis coli tumor suppressors [61]. Similarly, to adrenergic and noradrenergic signaling, muscarinic cholinergic signaling has pro-tumorigenic effects in colorectal cancer by transactivating EGFR (reviewed in [62]). In addition, pro-tumorigenic cholinergic signaling is also triggered by conjugated secondary bile acids such as lithocholyltaurine [63].

An extensive body of evidence suggests that in PDAC, sympathetic signaling has a pro-tumorigenic effect while parasympathetic signaling has an anti-tumorigenic effect. Xiao et al. substantiated the pro-tumorigenic effects of sympathetic signaling in PDAC by showing that β_2_-AR regulates the expression of aldo-keto reductase family 1 member B (AKR1B1) in human pancreatic cancer cells and promotes their proliferation via the ERK1/2 pathway [64]. In contrast, cholinergic signaling directly and indirectly suppresses pancreatic tumorigenesis and cancer stemness via muscarinic receptors. The anti-tumorigenic effect of cholinergic muscarinic signaling is achieved in part through CHRM1 and downstream effectors MAPK/EGFR and (phosphoinositide 3-kinase) PI3K/AKT [46]. 

In prostate cancer, both sympathetic and parasympathetic signaling has pro-tumorigenic effects. β_2_-AR signaling promotes proliferation and migration of prostate cancer cells via β-arrestin 2-mediated increase in cAMP levels and ERK1/2 activation [65]. In regard to parasympathetic signaling, M_1_-AChR mediates prostate cancer cell migration and invasion through hedgehog signaling [66], M_3_-AChR activates actin cytoskeleton and the MAPK signaling pathways [67]. In addition, M_1_-AChR and M_3_-AChR receptors promote castration-resistant growth of prostate cancer through a focal adhesion kinase (FAK)-YAP signaling axis [68]. 

In breast cancer it was shown that the SNS via β_2_ adrenergic signaling increases the infiltration of macrophages into the tumor environment. The infiltrating macrophages induce the expression of pro-metastatic molecules: Tgfb, Mmp9, Vegf, Vcam1, Csf1, Arg1 and Ptsg2. Collectively, these changes increase the metastatic burden in a murine breast cancer model [69]. The anti-tumorigenic effect of the sensory nerve was described in a mouse model of breast cancer. Capsaicin-induced depletion of sensory neurons resulted in the downregulation of Caspase-7 (an executor of apoptosis) and a disintegrin and metalloproteinase domain-containing protein 10 (ADAM-10). The pro-tumorigenic effects of ADAM-10 downregulation are explained by the fact that ADAM-10 actually hydrolyzes substance P to growth-inhibitory products, so that the loss of ADAM-10 exhausts an important tumorigenic suppressor [70].

In contrast to epithelial solid malignancies in which data on sympathetic signaling converge on a pro-tumorigenic effect, sympathetic signaling seems to portend an anti-tumorigenic effect in hematological malignancies. Hanoun et al. reported that the disruption of β_2_ adrenergic signaling promotes leukemic bone marrow infiltration in vivo [71]. In addition, development of acute myeloid leukemia disrupts SNS nerves and the quiescence of Nestin+ niche cells, leading to an expansion of phenotypic mesenchymal progenitor and stem cells primed for osteoblastic differentiation, at the expense of hematopoietic stem cell-maintaining neural/glial antigen 2 positive (NG2+) periarteriolar niche cells. The disruption of bone marrow adrenergic innervation is also responsible for chemotherapy-induced impairment of hematopoiesis [72]. In regard to parasympathetic signaling, in vitro data suggest that cholinergic signaling has anti-tumorigenic effects in chronic myelogenous leukemia cell lines through nicotinic and muscarinic type cholinergic receptors [73,74]. 

Similarly, to adrenergic signaling, a duality between solid epithelial and hematological malignancies seems to exist in regard to glucocorticoids, which are another class of major stress hormone. Glucocorticoids are potent anti-tumorigenic agents in hematological malignancies while in solid malignancies the effect is dependent on cancer type [75]. In fact, potent synthetic glucocorticoids such as prednisone and dexamethasone are often part of chemotherapy regimens in hematological malignancies (in particular lymphoid malignancies) (reviewed in [76]). 

Emerging evidence suggests that the nerve-tumor relationship recapitulates developmental programs of organogenesis. The morphogenesis of the embryonic submandibular gland is one of the most studied ex vivo system which captures the key relations between developing organs and nerves and which offers unique insights into the molecular mechanisms taking place within tumors [77]. The submandibular salivary gland achieves the necessary functional surface of secretory epithelium through a series of branching and budding orchestrated by the relation between the developing organ and nerves. Thus, the embryonic epithelial mesenchyme recruits nerves by secreting neurotrophic factors. In mammals, the neutrophin family has four members: nerve growth factor (NGF), which is the prototype neurotrophyn [78], brain-derived neurotrophic factor (BDNF), neurotrphin-3 (NT-3) and NT-4. The neutrotrophin family of NGF has both a high affinity receptor (tropomyosin-related kinase A (TRKA)) and a low-affinity p75 neurotrophin receptor (p75NTR) [79]. Another important family of neurotrophic factors is glial cell-derived neurotrophic factor (GDNF) family which includes artemin, neurturin (NRTN) and persephin, the cognate receptor being GDNF family receptor (GFR). 

During the embryologic development of the submandibular gland, the epithelial end-buds and ducts secrete neurturin, which binds neuronally expressed GFRα2 and triggers axonal outgrowth from the parasympathetic submandibular ganglion [80]. Parasympathetic nerves in turn release acetylcholine, which activates SRY-box 2 (SOX2)+ epithelial progenitors through muscarinic receptors, inducing acinar bud branching and maturation [81]. Similar mechanisms of gland development involving both PSNS and SNS have been described in the developing pancreas and limbs as well as during limb regeneration [82,83,84,85].

A growing body of evidence suggests that tumors coopt nerves for recapitulating the developmental programs of organogenesis. Thus, cancer cells establish a cancer-promoting feedback loop by stimulating axonogenesis or neuritogensis within the TME. In fact, a series of observational studies suggest that cancer cells are able to release neurotropic factors that stimulate axonal sprouting [36]. For instance, GDNF and other members of the GDNF family (artemin, NRTN and persephin) were shown to play important cancer promoting roles in pancreatic cancer [86]. Wang et al. observed that pancreatic cancer cells synthesize and release neurturin. Simultaneously, it was observed that the nerve fibers around tumor cells have an increased expression of GFRα-2, the specific receptor for NRTN. The tissue immunoreactivity for GFRα-2 directly correlated with a more severe pain phenotype in pancreatic cancer patients. In vitro matrigel-based invasion assay showed that NRTN enhances the invasiveness of pancreatic cancer cells and that it induces neuritogenesis of ex vivo dorsal root ganglions [87]. Similarly, Ceyhan et al. showed that pancreatic cancer and the surrounding non-cancerous pancreatic tissue exhibit an increase in nerve density compared to normal pancreatic tissue from control subjects. NGF and artemin expressions significantly correlated with neural hypertrophy and density. Finally, the authors showed that tissue extracts from pancreatic cancer as well as from the surrounding normal pancreatic tissue stimulate neuritogenesis in myenteric plexus-cultures, an effect which was abrogated by depletion of artemin and/or NGF [88]. 

A cancer promoting feedback loop by which cancer cells coopt nerves within the TME, which in turn paves the way for cancer growth and invasion was also described in prostate and in gastric cancers. Hayakawa et al. studied the interplay between nerves and gastric cancer cells and discovered that the main acetylcholine source in gastric cancer are nerve cells and chemosensory Tuft cells from gastric crypts. In turn, acetylcholine produced by these cells up-regulates NGF in cancer cells. In a feedback loop mechanism, NGF further increases nerve growth which promotes tumorigenesis. Mechanistically, it seems that acetylcholine stimulates tumor growth via its muscarinic acetylcholine receptor-3, activating YAP, a known modulator of Wnt/β-catenin signaling [59]. Prostate cancer cells contain higher levels of the precursor of NGF (proNGF) compared with benign hyperplastic cells. Clinically, the expression of proNGF significantly correlated with grade (i.e., the Gleason score) and further functional studies revealed that prostate cancer cell lines co-cultured with neurons can induce neurite outgrowth [89]. Interestingly, Dobrenis et al. highlighted the link between immune system and nerves in tumor progression. Granulocyte colony-stimulating factor (G-CSF) does not only stimulate the myeloid lineage, but it is also a neurotropic growth factor. In prostate cancer, G-CSF promotes the growth of both sympathetic and parasympathetic nerve fibers in the TME and the nerves further increase the metastatic potential of this tumor type [90]. Although the downstream effectors which mediate nerve-induced cancer growth are still being explored, these observational studies demonstrate that cancer hijacks developmental programs involving the autonomous nervous system.

In a seminal paper by Claire Magnon and collaborators [91], the authors discovered that *de novo* functional neurons from the subventricular zone of the central nervous system migrate through the blood and infiltrate the tumor stroma or metastatic tissue of prostate cancer, where they differentiate into adrenergic neurons. Thus, the authors described the presence within the TME of prostate cancer of nerve cells expressing doublecortin (DCX+), which is a classical marker of neural progenitors from the central nervous system. The high density of DCX+ cells are associated with an unfavorable outcome. In the periphery, DCX+ progenitor cells are capable to stimulate tumor initiation, tumor growth, and metastasis of prostate cancer cells [91].

### 2.2. Exosomes Are Key Components of the Communication between Nerve and Cancer Cells

The role of exosomes in the crosstalk between tumor cells and the nerves within the TME started from the observation that head and neck cancers are intensely innervated by autonomous sensory nerves and the degree of innervation is associated with decreased survival. Next, the authors employed a rat pheochromocytoma cell line, as an in vitro assay of neuritogenesis and observed that plasma exosomes from cancer patients or exosomes derived from tumor cells induced a significant neurite outgrowth while plasma exosomes from healthy donors or tonsil exosomes had a limited capacity to induce neurite outgrowth. Furthermore, in a series of elegant in vivo experiments, it was confirmed that tumor exosomes can induce neurite outgrowth. Mechanistically, the authors showed that the induction of neurite outgrowth by exosomes was not dependent on either NGF or BDNF, NT-3, NT-4 or GDNF. Instead, the authors discovered that erythropoietin-producing human hepatocellular (Eph) receptor-interacting proteins B1 (EphrinB1) packed into exosomes potentiated the growth of peritumoral nerve fibers. EphrinB1 is an axonal guidance molecule with important function in embryonic development that has the capacity to redirect axonal trajectory via the Ehp receptor. Importantly, the neuritogenesis-inducing capacity of exosomes from EphrinB1 null cancer cells is not completely abolished, suggesting that neuritogenesis induction takes place through a yet to be discovered mechanism. Nonetheless, the authors provided evidence that the process is dependent on MAP kinase signaling. Finally, the authors extended their observations in colorectal cancer, breast cancer, and melanoma, suggesting that exosome-mediated neurite outgrowth is important across cancer types [92]. In a subsequent study, the authors reported a similar exosome-based cancer-nerve communication operating in the case of cervical carcinoma [93]. 

Additional evidence linking exosomes to neurite outgrowth was provided by Ching et al., who showed that RNA molecules are key players in this process. The authors isolated exosomes from primary Schwann cells and adipose-derived stem cells differentiated towards a Schwann cell phenotype (dADSC) and observed that these exosomes were able to induce neurite outgrowth in vitro. When analyzing the exosome content, it was noticed that five miRNAs were overexpressed in exosomes from dADSC and in Schwann cells compared to undifferentiated stem cells: miR-18a, miR-182, miR-21, miR-222, and miR-1. Additionally, two mRNAs with important roles in neural growth were upregulated in exosomes from dADSC: *GAP43* and *Tau*. The authors hypothesized that these RNA molecules are responsible for the neurite outgrowth induced by exosomes from Schwann/Schwann-like cells [94].

In a recent publication, we characterized the crosstalk between head and neck cancer cells and the nerve fibers of the peripheral nervous system. We observed that loss of p53 induces a significant increase in nerve fibers in the TME and this phenomenon is associated with an unfavorable outcome. In an attempt to explore the mechanism that induces neuritogenesis, we observed that the growth of neural filaments of dorsal root ganglia (DRG) is controlled by exosomes released from p53 null head and neck cancer cells and not by exosomes from p53 WT cells. After characterizing the content of the exosomes, we noticed that the EVs from p53 null cells were depleted of the p53 regulated miRNA, miR-34a, compared to those from p53 WT cells. Additionally, exosomes released from miR-34a-5p deficient p53 WT cells lost their capacity to inhibit neuritogenesis. Regarding the mechanism that stimulates the growth of nerve fibers, we showed that miR-21 and miR-324, which are up-regulated in exosomes from p53 null cells, were responsible of this stimulatory signal. Moreover, the exosomes from p53 null cells not only increased the number of nerve fibers, but also induced the reprogramming of sensory nerves. In vivo studies revealed that exosomes from p53 null cells were capable to transdifferentiate sensory nerves to adrenergic nerves which further promote the growth of the tumor, thereby forming a positive feedback mechanism [95]. 

Taken together, these data support the concept that nerve cells and nerve fibers play an important role in tumorigenesis and that exosome communication and their RNA content contribute to this cross-talk. 

## 3. Communication between Immune Cells and Cancer Cells

### 3.1. The Immunological Landscape of Tumors Dictates the Outcome of Cancer Patients

The immune system plays a crucial role in cancer onset and progression, and it can be exploited as a therapeutic strategy [96]. The advent of immunotherapy, which proved to be game changer in a number of immunologically “hot” cancer types such as melanoma [97] or lung cancer [98], have sparkled interest towards the possibility of extending their use on all cancer types. However, many tumors are intrinsically immunologically “cold” and fail to respond to immunotherapy. Thus, the goal is to understand the molecular mechanisms which distinguish immunologically ‘cold’ tumors and to find ways to rekindle the immunogenicity of the tumors. 

The quest towards understanding the molecular mechanism behind the impaired immune response in cancer starts with the evolution of the tumor. Indeed, the immune system imposes an evolutionary constraint on the tumor growth, altering its natural genotypic and phenotypic trajectory, a phenomenon termed immunoediting [99]. Transformed cells develop escape mechanisms of resisting the control of the immune system (i.e., tumor-dependent escape) [100]. Alternatively, tumors dampen immunity by imposing an immunosuppressive state within the TME (i.e., immune system-dependent escape) [101]. Importantly, both innate and adaptive immunity are the culprits for the failure of the immune system to keep under control the malignant growth and for the establishment of an unfavorable immune landscape.

### 3.2. Communication between Tumor Cells and Adaptive Immunity Is both Direct and Exosome-Mediated

Adaptive immunity has been the focus of immunotherapy due to the immense success of chimeric antigen receptor T cells (CAR-T cells) and check point inhibitors in the treatment of a subset of cancer types with favorable immune landscape. For instance, the five-year survival of advanced melanoma patients treated with a combination of ipilimumab and nivolumab exceeded 50% [102]. The number of therapeutic strategies leveraging the immune system has expanded tremendously in the past years. For the moment, approved immunotherapies include the oncolytic virus talimogene laherparepvec for melanoma [103], the dendritic vaccine Sipuleucel-T for prostate cancer [104], two CD19 targeting CAR-T cells for acute lymphoblastic leukemia (ALL) and diffuse large B-cell lymphoma (DLBCL) [105], and seven checkpoint inhibitors targeting either the PD1/PD-L1 or CTLA-4/B7-1/B7-2 axis (reviewed in [106]). Additional forms of immunotherapy are currently under investigation.

The activation of the immune system must intrinsically limit itself to prevent triggering autoimmunity. This state of balance is often referred to as Th1/Th2 paradigm, which states that Th1 cells promote a proinflammatory phenotype and Th2 cells orchestrate an immunosuppressive phenotype [107]. In cancer, the equilibrium is aberrantly tilted towards the immunosuppressive phenotype and the goal of immunotherapy is to reverse it. The main mechanism of maintaining immunological homeostasis is based on direct cell-to-cell interaction. 

In the activation of T cells, the key molecular event is represented by the engagement of the T-cell receptor (TCR) by the processed antigen displayed on the MHC-I/II molecules. However, this event by itself is not enough for T cell activation, and the process is regulated by several co-stimulatory and co-inhibitory molecular signals (generally termed co-signaling). The B7-1/B7-2-CD28 is the prototype co-stimulatory axis in TCR signaling [108], while PD1-PD-L1/2 and CTLA-4 are the most well understood co-inhibitory axes [109,110]. The engagement of CD28 expressed on T cells by B7-1 (CD80) and B7-2 (CD86) on antigen presenting cells (and other cells) results in downstream activation of the PI3K and AKT signaling pathways which drive T cell activation [111,112]. In addition, CD28 also mediate some of the activating effect resulting from TCR engagement. Importantly, CTLA-4 axis operates mainly at sites of T-cell priming (e.g., secondary lymphoid organs), where B7/CD28-mediated co-stimulation is also prominent. In contrast, the PD1-PD-L1/2 axis operates mostly in non-lymphoid tissues and dampens T cell activation in the periphery [113].

Besides direct cell-to-cell communication, exosomes and EVs are emerging as key factors modulating adaptive immunity within the TME [114]. Maybruck et al. showed that head and neck cancer cells induce a suppressor phenotype in human CD8 T-cells via an exosomal immunomodulatory protein, galectin-1 (Gal-1) [115]. Exosomes derived from melanoma cells were shown to be enriched for a subset of coding and non-coding RNAs, most notably mmu-miR-709. These exosomes altered mitochondrial respiration and upregulated genes associated with the Notch signaling pathway in cytotoxic T cells [116].

### 3.3. Exosomes Are Key Components of the Communication between Innate Immunity and Cancer Cells

Besides adaptive immunity, it is becoming increasingly apparent that innate immunity plays a crucial role in determining the evolution of cancer. Importantly, innate and adaptive immunity are not independent but intertwined and an impaired innate immunity in the TME will ultimately result in aberrant adaptive immunity. Innate immunity cells governing the TME include macrophages, DCs, neutrophils, myeloid-derived stem cells (MDSCs), and natural killer cells (NKs). In each case, cell subtypes resembling the Th1/Th2 paradigm have been delineated. 

In the bloodstream, there are two major types of monocytes, classical “inflammatory” and non-classical, patrolling monocytes (PMo), performing pro-tumoral and anti-tumoral functions depending on cancer type/tissue of origin, differences in TME, stage of tumor growth and the experimental model and, most probably, on the tumor-secreted exosomes cargo [117,118]. Circulating monocytes extravasate into the tissue and differentiate into macrophages. Macrophages within the TME, termed tumor associated macrophages (TAMs), can compose up to 50% of the tumor mass [119]. Macrophages can be polarized into inflammatory M1 macrophages (classically activated) and M2 macrophages with immunosuppressive function (alternatively activated) [120]. Macrophages exert their main immune function by pathogen phagocytosis and antigen presentation, but also contribute to wound healing and tissue repair. Most TAMs are polarized towards the M2 phenotype and is associated with negative prognosis as well as treatment resistance [121]. The M2 phenotype is promoted by cytokines and hypoxia present in the TME. For instance, IL-4, abundantly present in the TME, promotes an M2 phenotype via STAT6 signaling (alternative activation) [122]. The wound healing and tissue repair functions of macrophages are exploited by the cancer cells to instead promote tumor growth, angiogenesis, and EMT [123].

Cancer-derived exosomes can induce both immunosuppressive or immunogenic phenotypes in surrounding monocytes and macrophages. For instance, exosomes secreted by non-aggressive, poorly metastatic melanoma block experimental lung metastasis by increasing the expansion of the PMo population in the lungs [124]. In support of the deleterious effect of cancer-derived exosomes, Kanlikilicer et al. unveiled a resistance mechanism to paclitaxel in ovarian cancer based on the exosomal transfer of oncogenic miR-1246 to M2-type macrophages, which results in the upregulation of multidrug resistance protein 1 (also called p-gp, MDR1 or ABCB1) [125]. Similarly, Cooks et al. showed that p53-mutant colon cancer cells, selectively shed miR-1246-enriched exosomes. When exosomes fuse with neighboring macrophages, the macrophage function is reprogrammed into a cancer promoting state with increased TGF-β activity [126]. 

Park et al. focused on the molecular mediators of tumor hypoxia across multiple tumor types [127]. They found that hypoxia induces the secretion of exosomes with the ability of promoting an M2-like phenotype and modifying the immunometabolic profile of infiltrating macrophages via let-7a miRNA, thus demonstrating a mechanism by which exosomal cargo allows tumor cells to influence the behavior of infiltrating immune cells [127].

The cross-talk between cancer cells and macrophages is bidirectional. In PDAC, macrophage-derived exosomes (MDE) significantly decreased the sensitivity of PDAC cells to gemcitabine, both in vitro and in vivo. This effect was mediated by transfer of oncogenic miR-365 to PDAC cells via exosomes, which resulted in upregulation of the triphosphonucleotide pool in cancer cells and the induction of the enzyme cytidine deaminase, whose effects is to inactivate gemcitabine [128]. Similarly, Challagundla et al. reported a mechanism of chemotherapy resistance in neuroblastoma (NBL) cells based on exosomal miRNAs exchanged between cancer cells and the neighboring monocytes [129]. The authors suggested that NBL cells transfer miR-21, which in turn upregulates miR-155 in monocytes through a TLR8 dependent mechanism and polarizes them towards an immunosuppressive M2 macrophage phenotype. MiR-155 is then transferred from monocytes to NBL cells via exosomes, resulting in the downregulation of TERF1, which in turn mediates resistance to cisplatin. This signaling circuitry seems to take place between NBL and monocytes but not between NBL and DC. The use of an inhibitor of exosome generation (GW4869), in fact, restores NBL cell drug sensitivity, even in the presence of surrounding monocytes, demonstrating the importance of exosomes as a potential therapeutic target [129].

MiRNA transfer between cancer cells and surrounding macrophages also takes place in a paracrine manner. Frank et al. provided evidence that miR-375 released by breast cancer cells during apoptosis could be involved in the modification of macrophages towards a tumor-supportive phenotype [130]. The results showed that macrophages take up miR-375 via CD36, which directly targets TNS3 and PXN enhancing macrophage migration and infiltration. In another study, Chen et al. found that miR-940, released via exosomes by ovarian cancer cells, targeted TAMs and promoted tumor growth via the CD206 and CD163 pathways [131].

MDSC are a heterogeneous population of immature macrophages, immature granulocytes, and immature DC [132]. MDSC present in the TME adopt an immunosuppressive phenotype, enhance tumor cell stemness, angiogenesis, and EMT, through IL-6 [133]. Glioma-derived exosomes (GDEs) influence the differentiation and activation of MDSC, as shown in a study centered on the role of GDEs in potentiating MDSC development [134]. GDEs induced a stronger MDSC expansion under hypoxic conditions, as compared to normoxia. Moreover, hypoxia induced miR-10a and miR-21 expression in GDE, which mediated MDSC expansion and activation by targeting RAR-related orphan receptor alpha (RORA) and phosphatase and tensin homolog (PTEN) pathway. In addition, GDE activate the pro-proliferative and immunosuppressive functions of MDSC in vitro and in vivo by exosomal transfer of miR-29a and miR-92a, which target high-mobility group box transcription factor 1 (Hbp1) and protein kinase cAMP-dependent type I regulatory subunit alpha (Prkar1a) [135]. 

NKs are circulatory innate lymphoid cytotoxic effector cells which eliminate cancer cells and limit metastases using death receptor-mediated apoptosis and perforin/granzyme-mediated cytotoxicity [136]. Consequently, low NK cell activity is associated with an increased cancer risk [137]. The cancer-killing capacity of NKs is impaired within the TME because of soluble factors and membrane encapsulated molecules [138]. Trying to understand how the TME-associated factors contribute to a drug-resistant phenotype in NBL, Neviani et al. described the role of NKs’ exosomes in the interplay between cancer cells and the TME. They showed that the cytotoxicity of NK exosomes depends not only on the presence of canonical killer molecules (i.e., perforin 1, granzyme A and B), but also on their nucleic acid cargo. In high risk NBL, the tumor suppressor miR-186, present in NK-derived exosomes, is downregulated. Exosomal delivery of miR-186 to NBL and NK cells impaired the survival and migration of NBL cells both in vitro and in vivo. Moreover, modulation of miR-186 abundance in these exosomes altered the NKs’ cytotoxic potential, suggesting that this miRNA may be at least in part responsible for their cytotoxic activity, highlighting the therapeutic potential of NK-derived exosomes in overcoming tumor growth [139].

DCs are antigen presenting cells (APCs) which bridge the gap between adaptive and innate immunity. DC present in the TME are referred to as tumor-infiltrating DC (TIDC). DC are broadly classified into classical, plasmacytoid DC, and monocyte-derived inflammatory DC [140]. DC and TIDC exhibit phenotypic plasticity and can both have immunogenic and immunosuppressive functions. Thus, TIDS are associated with a positive prognosis in endometrial carcinoma but a negative prognosis in breast cancer [141,142]. In addition, the immunosuppressive phenotype of TIDS usually accompanies more advanced forms of cancer. The culprits for polarizing TIDC towards an immunosuppressive phenotype include factors such as VEGF, IL-10, TGF-beta, and PGE2, leading to the activation of the indoleamine-pyrrole 2,3-dioxygenase (IDO), arginase 1 (Arg1), inducible nitric oxide synthase (iNOS), and STAT3 pathways and tilting the equilibrium towards Th2 activation [119]. Being professional APCs, DCs express a wide range of TLRs and cytokines, which play an important role in activation of immune response. However, though stimulation from the TME, DCs shift from effective antigen presenting cells into negative modulators of immune responses. In order to study the impact of exosomes on TLR4 in dendritic cells, Zhou et al. performed a transfection experiment with miR-203 mimics and inhibitors on a pancreatic cell line, as miR-203 was previously shown to be upregulated in PDAC. The results showed that miR-203 mimics could lead to a downregulation of TLR4 compared with the control group, while miR-203 inhibitors could reverse the downregulation of TLR4, thus suggesting that tumor-derived exosomes interfere with DCs via miR-203 [143].

In summary, communication between all types of immune and cancer cells and between nerve and cancer cells exists, and exosomes and their miRNA cargos (Table 3) which could be exploited as a potential therapeutic target.

## 4. Is There any Communication between Nerves and Immune Cells in the Tumor Microenvironment?

**Figure 1:**
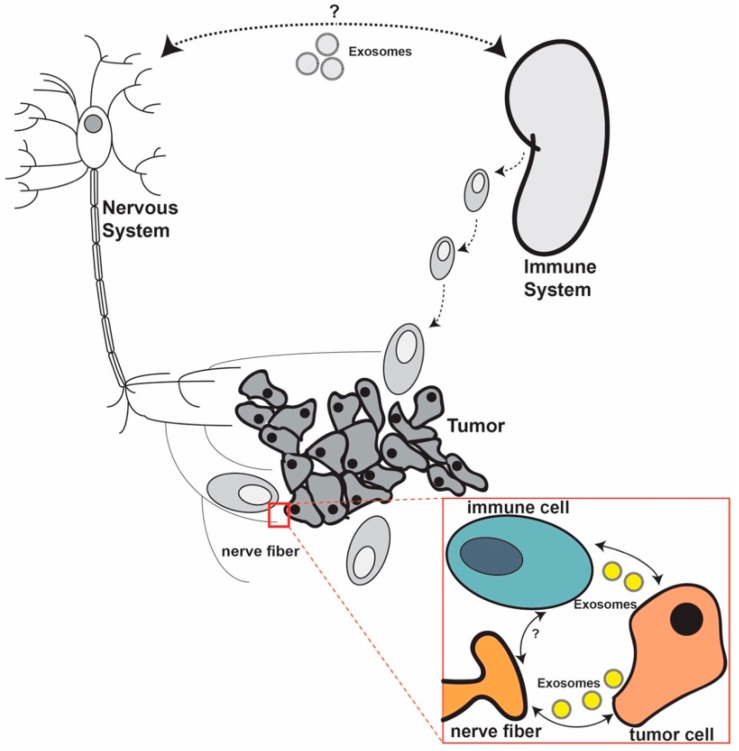
**We hypothesize that a direct crosstalk between nerves and immune cells in cancer exists.** This crosstalk might occur both at the systemic and within the tumor microenvironment (TME) (red box) levels. At a systemic level, the crosstalk may be mainly mediated by neurotransmitters and cytokines, but molecular mechanisms are needed to be further examined. At the TME level, the interplay between the three components can be either direct, through ligands, or indirect. We speculate that exosomes play an important role in this communication both at the systemic level and in the TME.

We hypothesize that the complex nerve–immune cell interplay takes place both within the TME (peripheral connection) but also at a systemic level (central connection) (Figure 1). Not much evidence regarding this interplay exists, but we speculate that exosomes, because of their proven role in the bilateral communication between cancer cells and nerves and between cancer cells and immune cells, might also serve as a communication vehicle between nerves and immune cells.

A triple interaction in the periphery, between nerves, blood vessels, and tumor cells, has been recently unveiled. Zahalka et al. showed that in prostate cancer adrenergic nerve fibers secreting noradrenalin stimulate the endothelial beta-adrenergic receptor and induce angiogenesis, supporting tumor growth. Mechanistically, noradrenalin signaling induces a metabolic switch in endothelial cells, promoting glycolytic metabolism which promotes angiogenesis [144]. A similar triple interaction in the TME could also exists between cancer cells, nerves and immune cells. Initial evidence in support of such a mechanism comes from Mo et al., who reported the expression of PD-L1 on peripheral nerve fibers from the stroma of prostate cancer using a novel monoclonal antibody against PD-L1. The authors observed that PD-L1 was positively expressed on the nerve branches of 69 out of 73 primary prostate cancers and that PD-L1 positive nerves were mainly localized in the peritumoral benign tissue. Moreover, the PD-L1 molecules expressed by nerves seemed to be functional, as the authors reported a negative correlation between the density of PD-L1 positive nerves and the CD8 tumor associated lymphocytes. Regarding the clinical implication of PD-L1 on tumor-associated nerves, it seems that a higher density of the ligand is associated with higher Gleason score, increased PNI, and poor prognosis [145].

Cavel et al. showed on pathological specimens from patients with PDAC that nerves invaded by cancer exhibit a high degree of infiltration by CD-68-positive macrophages [146]. The authors were able to establish a paracrine feedback loop between PDAC and endoneurial macrophages which drives PNI. PDAC cells recruit endoneurial macrophages by secreting CSF-1, which binds its receptor CSF-1R on macrophages, resulting in the polarization of macrophages towards a mixed M1/M2 phenotype via ERK signaling. In turn, activated endoneurial macrophages secrete GDNF, which induces PDAC invasion via the GDNF receptor GFRα1 expressed on PDAC cells, MEK-1 and PI3K signalling being key players in this process.

In PDAC, it was observed that PNI is linked to lower levels of CD8 T cells and Th1 cells and increased levels of Th2 cells, creating a pro-tumorigenic TME. Mechanistically, nerves invaded by cancer cells secreted more acetylcholine in the TME. Acetylcholine has a broad role on the immune phenotype, supporting the differentiation of Th2 cells in favor of Th1, inhibiting the recruitment of CD8 cells and decreasing the levels of IFN-gamma. These results were further confirmed in a murine model of sub-diaphragmatic bilateral vagotomy, which resulted in inhibited PNI and tumor growth, increased Th1 and CD8 cell density, inhibited tumor growth, and prolonged OS [147]. Similarly, it was noticed that in PDAC, PNI is induced by bone marrow macrophages expressing GDNF ligand that stimulates tumor cell dissemination along nerves via activating RET [148].

The direct communication between immune cells and nerves via exosomes in the TME has not yet been described. On the other hand, this type of communication was detected between sensory neurons of DRGs and macrophages after nerve injury and this phenomenon partially explains neuropathic pain. Simeoli et al. have shown that sensory neurons, upon activation, release exosomes enriched in miR-21-5p. The exosomes containing miR-21-5p are taken up by macrophages inducing a pro-inflammatory switch. In vivo data further strengthen these findings, as mice with nerve injuries showed an upregulation of miR-21-5p while the inhibition of the same miRNA reduced neuropathic pain and recruitment of macrophages with inflammatory phenotype [149].

In summary, these findings make us confident that in cancer, a direct communication between nerves and immune cells exists and that this is mediated by exosomes. Future studies are necessary to further describe this interaction.

We also hypothesize that communication between the nervous and the immune systems outside the TME could influence the oncogenic process. Only limited evidence of this interaction exists. For example, Frick et al., starting from the concept that stress affects the immune system, observed that chronic stress alters the T-cell immunity in lymphoma mice. Chronically stressed mice with lymphoma displayed a reduced T-cell proliferation, lower numbers of CD4 lymphocytes, and lower levels of TNF-alpha and IFN-gamma. Furthermore, these mice showed an increased proliferation rate of cancer cells and shorter OS compared to unstressed mice [150]. Although the authors did not delve into the molecular mechanism underlying this observation, it is likely that stress-related immunosuppression drives cancer progression via neurotransmitters and even EVs. Indeed, immune cells express β_2_-AR and catecholamine-mediated signaling can regulate their function and tumor immune responses. Norepinephrine secreted by postganglionic sympathetic neurons innervating secondary lymphoid organs can decrease IFN-gamma and TNF-alpha secretion by primary human CD8+ effector memory T cells and suppress their cytolytic capacity [151]. Blocking the beta-adrenergic signaling proved to be effective in improving the efficacy of anti-tumor vaccine by enhancing the frequency of CD8+ T lymphocytes infiltrating the tumor (TIL). This positive effect mainly occurs in the tumor-draining lymph node during the initial phase of antitumor CD8+ T-cell priming [152]. The immunosuppressive potential of MDSCs is also regulated by β_2_ adrenergic receptor–mediated signaling. In particular, β_2_ adrenergic signaling regulates MDSC frequency and survival in tumors, and modulates the expression of two important immunosuppressive molecules, such as PD-L1 and arginase-I, resulting in increased suppression of T cell functions [153]. These evidences further confirm the importance of neuronal infiltration in the regulation of tumor immune responses in the TME. Beside local delivery of neurotransmitters by tumor-associated neurons, adrenergic signaling can be mediated by increased norepinephrine systemic levels. In the experimental autoimmune encephalomyelitis model of multiple sclerosis, β_2_-AR–mediated signaling was able to reduce the T cell-mediated autoimmunity in experimental autoimmune encephalomyelitis by suppressing IL-2, IFN-gamma, and GM-CSF production [154]. This evidence demonstrates the relevant effect of chronic stress in cancer patients, as it can be associated with increased systemic levels of catecholamine and in turn affects tumor immune responses.

Nonetheless, other mechanisms like exosomes and EVs can be implicated and need to be further researched. More recently, Cheng et al., discovered that prostate cancer tumorigenesis is accelerated by chronic depression in vivo. Depressed mice had more infiltrating TAMs, which were recruited from the spleen and from circulating MDSC. Furthermore, the authors noticed that the stress hormone norepinephrine stimulated cancer cells to secrete neuropeptide Y, which further activated myeloid cells mobilization. In clinical samples, depressed patients had a higher density of TAMs and higher levels of neuropeptide Y [155].

If this hypothesis is confirmed, it will shed new light on our understanding of cancer as a systemic disease. Epidemiologic data has already linked psychological trauma with cancer progression and unfavorable outcomes. In a systematic review analyzing 165 publications, it was proven that stress-related psychosocial factors increase the incidence of cancer while the analysis of another 330 studies linked stress factors with poor survival [156]. Hence, understanding the systemic connection between the immune system and the nervous system will further clarify how behavioral factors like isolation, depression, and stress play a mechanistic role in tumorigenesis.

## 5. Future Perspectives

All these data together lead to an important novel therapeutic perspective: simultaneously inhibiting the nerve and the immune axis that promote tumor progression. In other words, combining nervous stimulatory signaling blockade with immune checkpoint inhibitors may be an alternative way to treat cancer. Moreover, one should also take into consideration the possibility to manipulate the exosome trafficking between these cells in order to inhibit tumor growth.

The main method to restore the immune system in cancer is immune checkpoint inhibition. Immune checkpoint inhibitors have expanded treatment options for cancer patients with unfavorable outcomes; however, an important limitation exists since only a small proportion of patients respond to the therapy [157]. Therefore, alternative approaches need to be discovered to increase the proportion of patients that could positively respond to immune checkpoint inhibitors. One solution would be the inhibition of the molecular crosstalk between nerve and tumor cells. We briefly discuss below several potential strategies that could accomplish this aspiration.

One of the simplest methods to block the pro-oncogenic signaling of nerves could be the use of beta-blockers. In fact, numerous clinical trials are already assessing the efficacy of beta-blockers in different cancers [36]. Alternatively, tumors could be decoupled from the pro-oncogenic signal of nerves by performing surgical or chemical denervation (see Section 2.1).

The suppression of nerve recruitment within the TME and the inhibition of the reprograming of nerves towards a pro-tumorigenic phenotype by cancer cells represents another possible strategy to improve cancer outcomes. For instance, neurotrophic factors, like NGF, could be targeted using monoclonal antibodies or siRNAs against them. Bapat et al. used anti-NGF siRNAs in pancreatic cancer cell lines and observed that the migration of the cells towards DRGs was inhibited. Additionally, rat pheochromocytoma cells (PC-12) cultured with conditioned media from pancreatic cancer cell lines with NGF knock-down showed reduced neurite outgrowth [158].

Yet another option to block the pro-oncogenic signaling of nerves might be to stop exosome production in cancer cells. We have showed that blocking the exosome secretion pathway by double knock-out of RAB27A/RAB27B suppresses the ability of head and neck cancer cells to induce the trans-differentiation of sensory nerves into adrenergic nerves, resulting in suppressed tumor growth [95].

Moreover, the efficacy of combining immune checkpoint inhibitors with beta-blockers is supported by epidemiologic data and pre-clinical experiments. By analyzing 195 patients with melanoma receiving immunotherapy (either IL-2, anti-CTLA-4 and/or anti-PD1), it was remarked that the addition of pan-beta-blockers was associated with a significantly better OS compared to patients receiving immunotherapy plus beta1-blockers or no beta-blockers. By performing in vivo experiments, it was confirmed that the addition of propranolol (non-selective beta-blocker) improved the survival of mice receiving anti-PD1 or anti-PD1 and IL-2, but not of mice receiving IL-2 alone. In fact, beta-blockers alone are enough to boost the efficacy of immune-based therapies in mice [159]. However, the authors did not provide any additional results regarding the molecular mechanisms of action.

Thus, the crosstalks between nerves and cancer cells and between immune and cancer cells seem to intersect and novel therapies need to be developed to simultaneously inhibit these synergistic mechanisms. Compelling evidence suggest that exosomes play an important role in these communication avenues and targeting exosomes or modulating their content could be an innovative strategy to move this concept into clinical practice.

## Figures and Tables

**Table 1:** Summary of adrenergic and cholinergic receptor subtypes, effectors and ligands.

Receptor	Subtype	Effector	Ligand
Adrenergic receptors (AR)	alpha 1	G_q_: ↑PLC, ↑PIP_3_, ↑DAG, ↑Ca^2+^	adrenaline, noradrenaline
	alpha 2	G_i_: ↓AC, ↓cAMP	adrenaline, noradrenaline
	beta 1	G_s_: ↑AC, ↑cAMP	adrenaline, noradrenaline
	beta 2	G_s_: ↑AC, ↑cAMP	adrenaline, noradrenaline
	beta 3	G_s_: ↑AC, ↑cAMP	adrenaline, noradrenaline
Cholinergic receptors	Nicotinic	↑Na^+^, ↑K^+^	acetylcholine, nicotine
	Muscarinic M_1_	G_q_: ↑ PLC, ↑PIP3, ↑DAG, ↑Ca2+	acetylcholine, muscarine
	Muscarinic M_2_	G_i_: ↓AC, ↓cAMP	acetylcholine, muscarine
	Muscarinic M_3_	G_q_: ↑ PLC, ↑PIP_3_, ↑DAG, ↑Ca^2+^	acetylcholine, muscarine
	Muscarinic M_4_	G_i_: ↓AC, ↓cAMP	acetylcholine, muscarine
	Muscarinic M_5_	G_q_: ↑ PLC, ↑PIP_3_, ↑DAG, ↑Ca^2+^	acetylcholine, muscarine

Gi = G-protein alpha subunit, group S; G_s_ = G-protein alpha subunit, group S; G_q_ = G-protein alpha subunit, group Q; PLC = phospholipase C; AC = adenylate cyclase; DAG = diacylglycerol; PIP_3_ = phosphatidylinositol 3,4,5 trisphosphate.

**Table 2:** The relationship between cancer type and the effect of sympathetic and parasympathetic innervation.

Cancer Type	Sympathetic Innervation	Parasympathetic Innervation	References
Lung Cancer	Pro-tumorigenic	Pro-tumorigenic	[52,53,54,55]
Gastric cancer	Pro-tumorigenic	Pro-tumorigenic	[47,56,57,58,59]
Colorectal Cancer	Pro-tumorigenic	Pro-tumorigenic	[60,61,62,63]
Pancreatic ductal adenocarcinoma	Pro-tumorigenic	Anti-tumorigenic	[46,64]
Prostate cancer	Pro-tumorigenic	Pro-tumorigenic	[65,66,67,68]
Breast cancer	Pro-tumorigenic	Anti-tumorigenic	[69,70]
Hematological malignancies	Anti-tumorigenic	Anti-tumorigenic	[71,72,73,74]

**Table 3:** Exosomes as a communication tool between nerves and cancer cells and between immune and cancer cells, and the consequently generated effects.

Exosome Origin	Target Cells	Key Molecules Involved	Effect	Ref
Head and neck squamous cell carcinoma cell lines (other cancers: CRC, BC, melanoma)	PC12 cells (rat pheochromocytoma cell line)	EphrinB1 protein	Neurite outgrowth (in vitro) and tumor innervation (in vivo).	[92]
dADSC, primary Schwann cells	NG108–15 neurons	miRNAs: miR-18a, miR-21, miR-182, miR-222, miR-1, and mRNAs: *GAP43* and *Tau*	Neurite outgrowth.	[94]
p53 null head and neck cancer cells	Peritumoral nerve fibers, DRGs and TGs	Low levels of miR-34a and high levels of miR-21 and miR-324	Neurite outgrowth and transdifferentiation of sensory neurons in adrenergic neurons.	[95]
Head and neck cancer cells	CD8+ T cells	Galectin-1 (immunoregulatory protein)	Stimulation of CD8+ T-cell suppressor phenotype.	[115]
Melanoma cell lines	CTLL2 Cytotoxic T cell lines	miR-709, miR-2137, miR-2861, miR-1195, miR-762 (the five most highly abundant miRNAs)	Transcriptome signature changes resulting in mitochondrial respiration alteration.	[116]
Poorly metastatic melanoma cells	Patrolling monocytes (PMo)	Nr4a transcription factor and pigment epithelium-derived factor	PMo conditioned innate immune response with cancer cell clearance at the metastatic niche.	[124]
Neuroblastoma cell lines	Monocytes	miR-21	Protumoral activity of monocytes through miR-21/TLR8-NF-кB/exosomic miR-155/TERF1 signaling pathway.	[129]
Ovarian cancer cell lines	Macrophages	miR-1246	Transfer of oncogenic miR-1246 to M2-type macrophages, but not M0-type macrophages.	[125]
p53 mutant CRC cells	Macrophages	miR-1246	Macrophage miR-1246-dependent reprogramming into a cancer promoting state with increased TGF-beta activity.	[126]
Melanoma cell lines	Macrophages	let-7a	Macrophage increased oxidative phosphorylation activity and M2-like polarization.	[127]
Glioma cell lines under hypoxic conditions	MDSC	miR-10a and miR-21	Hypoxia-inducible expression of miR-10a and miR-21 mediates MDSC expansion and activation by targeting RORA and PTEN.	[134]
Glioma cell lines	MDSC	miR-29a and miR-92a	MDSC expansion and function activation through miRNA-29a/Hbp1 and miRNA-92a/Prkar1a pathways.	[135]
Pancreatic adenocarcinoma cell lines	DC	miR-203	Downregulation of TLR4 and downstream cytokines.	[143]

dADSC—Adipose derived stem cells were differentiated towards a Schwann cell-like phenotype; MDSCs—Myeloid-derived suppressor cells; DC—Dendritic cells; DRG—dorsal root ganglia; TG—trigeminal ganglia; CRC—colorectal cancer; BC—breast cancer; PMo—patrolling monocytes; RORA, RAR-related orphan receptor alpha; PTEN, phosphatase and tensin homolog.

## References

[1] Dagenais G.R., Leong D.P., Rangarajan S., Lanas F., Lopez-Jaramillo P., Gupta R., Diaz R., Avezum A., Oliveira G.B.F., Wielgosz A. (2020). Variations in common diseases, hospital admissions, and deaths in middle-aged adults in 21 countries from five continents (PURE): A prospective cohort study. Lancet.

[2] Hanahan D., Weinberg R.A. (2011). Hallmarks of cancer: The next generation. Cell.

[3] Zitvogel L., Tesniere A., Kroemer G. (2006). Cancer despite immunosurveillance: Immunoselection and immunosubversion. Nat. Rev. Immunol..

[4] Amit M., Na’ara S., Gil Z. (2016). Mechanisms of cancer dissemination along nerves. Nat. Rev. Cancer.

[5] Chen D.S., Mellman I. (2013). Oncology meets immunology: The cancer-immunity cycle. Immunity.

[6] Apte R.S., Chen D.S., Ferrara N. (2019). VEGF in signaling and disease: Beyond discovery and development. Cell.

[7] Coffelt S.B., Lewis C.E., Naldini L., Brown J.M., Ferrara N., De Palma M. (2010). Elusive identities and overlapping phenotypes of proangiogenic myeloid cells in tumors. Am. J. Pathol..

[8] Socinski M.A., Jotte R.M., Cappuzzo F., Orlandi F., Stroyakovskiy D., Nogami N., Rodriguez-Abreu D., Moro-Sibilot D., Thomas C.A., Barlesi F. (2018). Atezolizumab for first-line treatment of metastatic nonsquamous NSCLC. N. Engl. J. Med..

[9] Demir I.E., Friess H., Ceyhan G.O. (2012). Nerve-cancer interactions in the stromal biology of pancreatic cancer. Front. Physiol..

[10] Quail D.F., Joyce J.A. (2013). Microenvironmental regulation of tumor progression and metastasis. Nat. Med..

[11] Kalluri R., LeBleu V.S. (2020). The biology, function, and biomedical applications of exosomes. Science.

[12] Barcellos-Hoff M.H., Lyden D., Wang T.C. (2013). The evolution of the cancer niche during multistage carcinogenesis. Nat. Rev. Cancer.

[13] Peinado H., Aleckovic M., Lavotshkin S., Matei I., Costa-Silva B., Moreno-Bueno G., Hergueta-Redondo M., Williams C., Garcia-Santos G., Ghajar C. (2012). Melanoma exosomes educate bone marrow progenitor cells toward a pro-metastatic phenotype through MET. Nat. Med..

[14] Litwack G. (2015). Hormones and transport systems. Preface. Vitam. Horm..

[15] Raposo G., Stoorvogel W. (2013). Extracellular vesicles: Exosomes, microvesicles, and friends. J. Cell Biol..

[16] Cocucci E., Meldolesi J. (2015). Ectosomes and exosomes: Shedding the confusion between extracellular vesicles. Trends Cell Biol..

[17] Harding C., Heuser J., Stahl P. (1984). Endocytosis and intracellular processing of transferrin and colloidal gold-transferrin in rat reticulocytes: Demonstration of a pathway for receptor shedding. Eur. J. Cell Biol..

[18] Gurunathan S., Kang M.H., Jeyaraj M., Qasim M., Kim J.H. (2019). Review of the isolation, characterization, biological function, and multifarious therapeutic approaches of exosomes. Cells.

[19] Wubbolts R., Leckie R.S., Veenhuizen P.T., Schwarzmann G., Mobius W., Hoernschemeyer J., Slot J.W., Geuze H.J., Stoorvogel W. (2003). Proteomic and biochemical analyses of human B cell-derived exosomes. Potential implications for their function and multivesicular body formation. J. Biol. Chem..

[20] Colombo M., Raposo G., Thery C. (2014). Biogenesis, secretion, and intercellular interactions of exosomes and other extracellular vesicles. Annu. Rev. Cell Dev. Biol..

[21] Jenjaroenpun P., Kremenska Y., Nair V.M., Kremenskoy M., Joseph B., Kurochkin I.V. (2013). Characterization of RNA in exosomes secreted by human breast cancer cell lines using next-generation sequencing. PeerJ.

[22] Cortez M.A., Bueso-Ramos C., Ferdin J., Lopez-Berestein G., Sood A.K., Calin G.A. (2011). MicroRNAs in body fluids--the mix of hormones and biomarkers. Nat. Rev. Clin. Oncol..

[23] Pardini B., Calin G.A. (2019). MicroRNAs and long non-coding RNAs and their hormone-like activities in cancer. Cancers.

[24] De Los Santos M.C., Dragomir M.P., Calin G.A. (2019). The role of exosomal long non-coding RNAs in cancer drug resistance. Cancer Drug Resist..

[25] Dragomir M., Chen B., Calin G.A. (2018). Exosomal lncRNAs as new players in cell-to-cell communication. Transl. Cancer Res..

[26] Thery C., Ostrowski M., Segura E. (2009). Membrane vesicles as conveyors of immune responses. Nat. Rev. Immunol..

[27] Valadi H., Ekstrom K., Bossios A., Sjostrand M., Lee J.J., Lotvall J.O. (2007). Exosome-mediated transfer of mRNAs and microRNAs is a novel mechanism of genetic exchange between cells. Nat. Cell Biol..

[28] Vasilescu C., Tanase M., Giza D., Procopiuc L., Dragomir M.P., Calin A.G.A. (2020). How does a tumor get its shape? MicroRNAs act as morphogens at the cancer invasion front. Noncoding RNA.

[29] Nishida-Aoki N., Ochiya T. (2015). Interactions between cancer cells and normal cells via miRNAs in extracellular vesicles. Cell. Mol. Life Sci..

[30] Tkach M., Thery C. (2016). Communication by extracellular vesicles: Where we are and where we need to go. Cell.

[31] Josson S., Gururajan M., Sung S.Y., Hu P., Shao C., Zhau H.E., Liu C., Lichterman J., Duan P., Li Q. (2015). Stromal fibroblast-derived miR-409 promotes epithelial-to-mesenchymal transition and prostate tumorigenesis. Oncogene.

[32] Wehrwein E.A., Orer H.S., Barman S.M. (2016). Overview of the anatomy, physiology, and pharmacology of the autonomic nervous system. Compr. Physiol..

[33] Saloman J.L., Albers K.M., Li D., Hartman D.J., Crawford H.C., Muha E.A., Rhim A.D., Davis B.M. (2016). Ablation of sensory neurons in a genetic model of pancreatic ductal adenocarcinoma slows initiation and progression of cancer. Proc. Natl. Acad. Sci. USA.

[34] Moreno-Smith M., Lutgendorf S.K., Sood A.K. (2010). Impact of stress on cancer metastasis. Future Oncol..

[35] Kanczkowski W., Sue M., Bornstein S.R. (2016). Adrenal gland microenvironment and its involvement in the regulation of stress-induced hormone secretion during sepsis. Front. Endocrinol. (Lausanne).

[36] Faulkner S., Jobling P., March B., Jiang C.C., Hondermarck H. (2019). Tumor neurobiology and the war of nerves in cancer. Cancer Discov..

[37] Liebig C., Ayala G., Wilks J.A., Berger D.H., Albo D. (2009). Perineural invasion in cancer: A review of the literature. Cancer.

[38] Aurello P., Berardi G., Tierno S.M., Rampioni Vinciguerra G.L., Socciarelli F., Laracca G.G., Giulitti D., Pilozzi E., Ramacciato G. (2017). Influence of perineural invasion in predicting overall survival and disease-free survival in patients With locally advanced gastric cancer. Am. J. Surg..

[39] Skancke M., Arnott S.M., Amdur R.L., Siegel R.S., Obias V.J., Umapathi B.A. (2019). Lymphovascular invasion and perineural invasion negatively impact overall survival for stage II adenocarcinoma of the colon. Dis. Colon Rectum.

[40] Ayala G.E., Dai H., Ittmann M., Li R., Powell M., Frolov A., Wheeler T.M., Thompson T.C., Rowley D. (2004). Growth and survival mechanisms associated with perineural invasion in prostate cancer. Cancer Res..

[41] Sheng L., Ji Y., Du X. (2015). Perineural invasion correlates with postoperative distant metastasis and poor overall survival in patients with PT1-3N0M0 esophageal squamous cell carcinoma. Onco Targets Ther..

[42] Yokoyama S., Matsuda K., Watanabe T., Mitani Y., Ieda J., Iwamoto H., Hotta T., Takifuji K., Yamaue H. (2017). Perineural invasion is associated with poor survival after preoperative chemoradiation therapy for advanced lower rectal cancer. Dig. Surg..

[43] Murakami Y., Uemura K., Sudo T., Hashimoto Y., Kondo N., Nakagawa N., Muto T., Sasaki H., Urabe K., Sueda T. (2013). Perineural invasion in extrahepatic cholangiocarcinoma: Prognostic impact and treatment strategies. J. Gastrointest. Surg..

[44] Bakst R.L., Glastonbury C.M., Parvathaneni U., Katabi N., Hu K.S., Yom S.S. (2019). Perineural invasion and perineural tumor spread in head and neck cancer. Int. J. Radiat. Oncol. Biol. Phys..

[45] Partecke L.I., Kading A., Trung D.N., Diedrich S., Sendler M., Weiss F., Kuhn J.P., Mayerle J., Beyer K., von Bernstorff W. (2017). Subdiaphragmatic vagotomy promotes tumor growth and reduces survival via TNFalpha in a murine pancreatic cancer model. Oncotarget.

[46] Renz B.W., Tanaka T., Sunagawa M., Takahashi R., Jiang Z., Macchini M., Dantes Z., Valenti G., White R.A., Middelhoff M.A. (2018). Cholinergic signaling via muscarinic receptors directly and indirectly suppresses pancreatic tumorigenesis and cancer stemness. Cancer Discov..

[47] Zhao C.M., Hayakawa Y., Kodama Y., Muthupalani S., Westphalen C.B., Andersen G.T., Flatberg A., Johannessen H., Friedman R.A., Renz B.W. (2014). Denervation suppresses gastric tumorigenesis. Sci. Transl. Med..

[48] Magnon C., Hall S.J., Lin J., Xue X., Gerber L., Freedland S.J., Frenette P.S. (2013). Autonomic nerve development contributes to prostate cancer progression. Science.

[49] Zahalka A.H., Frenette P.S. (2020). Nerves in cancer. Nat. Rev. Cancer.

[50] Sinha S., Fu Y.Y., Grimont A., Ketcham M., Lafaro K., Saglimbeni J.A., Askan G., Bailey J.M., Melchor J.P., Zhong Y. (2017). PanIN neuroendocrine cells promote tumorigenesis via neuronal cross-talk. Cancer Res..

[51] Bai H., Li H., Zhang W., Matkowskyj K.A., Liao J., Srivastava S.K., Yang G.Y. (2011). Inhibition of chronic pancreatitis and pancreatic intraepithelial neoplasia (PanIN) by capsaicin in LSL-KrasG12D/Pdx1-Cre mice. Carcinogenesis.

[52] Nilsson M.B., Le X., Heymach J.V. (2020). Beta-adrenergic signaling in lung cancer: A potential role for beta-blockers. J. Neuroimmune Pharmacol..

[53] Nilsson M.B., Sun H., Diao L., Tong P., Liu D., Li L., Fan Y., Poteete A., Lim S.O., Howells K. (2017). Stress hormones promote EGFR inhibitor resistance in NSCLC: Implications for combinations with beta-blockers. Sci. Transl. Med..

[54] Friedman J.R., Richbart S.D., Merritt J.C., Brown K.C., Nolan N.A., Akers A.T., Lau J.K., Robateau Z.R., Miles S.L., Dasgupta P. (2019). Acetylcholine signaling system in progression of lung cancers. Pharmacol. Ther..

[55] Shao J.X., Wang B., Yao Y.N., Pan Z.J., Shen Q., Zhou J.Y. (2016). Autonomic nervous infiltration positively correlates with pathological risk grading and poor prognosis in patients with lung adenocarcinoma. Thorac. Cancer.

[56] Zhang X., Zhang Y., He Z., Yin K., Li B., Zhang L., Xu Z. (2019). Chronic stress promotes gastric cancer progression and metastasis: An essential role for ADRB2. Cell Death Dis..

[57] Zhi X., Li B., Li Z., Zhang J., Yu J., Zhang L., Xu Z. (2019). Adrenergic modulation of AMPKdependent autophagy by chronic stress enhances cell proliferation and survival in gastric cancer. Int. J. Oncol..

[58] Shi M., Yang Z., Hu M., Liu D., Hu Y., Qian L., Zhang W., Chen H., Guo L., Yu M. (2013). Catecholamine-Induced beta2-adrenergic receptor activation mediates desensitization of gastric cancer cells to trastuzumab by upregulating MUC4 expression. J. Immunol..

[59] Hayakawa Y., Sakitani K., Konishi M., Asfaha S., Niikura R., Tomita H., Renz B.W., Tailor Y., Macchini M., Middelhoff M. (2017). Nerve growth factor promotes gastric tumorigenesis through aberrant cholinergic signaling. Cancer Cell.

[60] Chin C.C., Li J.M., Lee K.F., Huang Y.C., Wang K.C., Lai H.C., Cheng C.C., Kuo Y.H., Shi C.S. (2016). Selective beta2-AR blockage suppresses colorectal cancer growth through regulation of EGFR-Akt/ERK1/2 signaling, G1-phase arrest, and apoptosis. J. Cell Physiol..

[61] Han J., Jiang Q., Ma R., Zhang H., Tong D., Tang K., Wang X., Ni L., Miao J., Duan B. (2020). Norepinephrine-CREB1-miR-373 axis promotes progression of colon cancer. Mol. Oncol..

[62] Xie G., Raufman J.P. (2016). Muscarinic receptor signaling and colon cancer progression. J. Cancer Metastasis Treat..

[63] Raufman J.P., Chen Y., Cheng K., Compadre C., Compadre L., Zimniak P. (2002). Selective interaction of bile acids with muscarinic receptors: A case of molecular mimicry. Eur. J. Pharmacol..

[64] Xiao M.B., Jin D.D., Jiao Y.J., Ni W.K., Liu J.X., Qu L.S., Lu C.H., Ni R.Z., Jiang F., Chen W.C. (2018). beta2-AR regulates the expression of AKR1B1 in human pancreatic cancer cells and promotes their proliferation via the ERK1/2 pathway. Mol. Biol. Rep..

[65] Zhang P., He X., Tan J., Zhou X., Zou L. (2011). beta-arrestin2 mediates beta-2 adrenergic receptor signaling inducing prostate cancer cell progression. Oncol. Rep..

[66] Yin Q.Q., Xu L.H., Zhang M., Xu C. (2018). Muscarinic acetylcholine receptor M1 mediates prostate cancer cell migration and invasion through hedgehog signaling. Asian J. Androl..

[67] Guo L., Liu Y., Ding Z., Sun W., Yuan M. (2016). Signal transduction by M3 muscarinic acetylcholine receptor in prostate cancer. Oncol. Lett..

[68] Goto Y., Ando T., Izumi H., Feng X., Arang N., Gilardi M., Wang Z., Ando K., Gutkind J.S. (2020). Muscarinic receptors promote castration-resistant growth of prostate cancer through a FAK-YAP signaling axis. Oncogene.

[69] Sloan E.K., Priceman S.J., Cox B.F., Yu S., Pimentel M.A., Tangkanangnukul V., Arevalo J.M., Morizono K., Karanikolas B.D., Wu L. (2010). The sympathetic nervous system induces a metastatic switch in primary breast cancer. Cancer Res..

[70] Erin N., Zhao W., Bylander J., Chase G., Clawson G. (2006). Capsaicin-induced inactivation of sensory neurons promotes a more aggressive gene expression phenotype in breast cancer cells. Breast Cancer Res. Treat..

[71] Hanoun M., Zhang D., Mizoguchi T., Pinho S., Pierce H., Kunisaki Y., Lacombe J., Armstrong S.A., Duhrsen U., Frenette P.S. (2014). Acute myelogenous leukemia-induced sympathetic neuropathy promotes malignancy in an altered hematopoietic stem cell niche. Cell Stem Cell.

[72] Lucas D., Scheiermann C., Chow A., Kunisaki Y., Bruns I., Barrick C., Tessarollo L., Frenette P.S. (2013). Chemotherapy-induced bone marrow nerve injury impairs hematopoietic regeneration. Nat. Med..

[73] Aydin B., Cabadak H., Goren M.Z. (2018). Investigation of the roles of non-neuronal acetylcholine in chronic myeloid leukemic cells and their erythroid or megakaryocytic differentiated lines. Anticancer Agents Med. Chem..

[74] Cabadak H., Aydin B., Kan B. (2011). Regulation of M2, M3, and M4 muscarinic receptor expression in K562 chronic myelogenous leukemic cells by carbachol. J. Recept. Signal Transduct. Res..

[75] Lin K.T., Wang L.H. (2016). New dimension of glucocorticoids in cancer treatment. Steroids.

[76] Pufall M.A. (2015). Glucocorticoids and Cancer. Adv. Exp. Med. Biol..

[77] Mattingly A., Finley J.K., Knox S.M. (2015). Salivary gland development and disease. Wiley Interdiscip. Rev. Dev. Biol..

[78] Aloe L., Rocco M.L., Balzamino B.O., Micera A. (2016). Nerve growth factor: Role in growth, differentiation and controlling cancer cell development. J. Exp. Clin. Cancer Res..

[79] Fielder G.C., Yang T.W., Razdan M., Li Y., Lu J., Perry J.K., Lobie P.E., Liu D.X. (2018). The GDNF family: A role in cancer?. Neoplasia.

[80] Knox S.M., Lombaert I.M., Haddox C.L., Abrams S.R., Cotrim A., Wilson A.J., Hoffman M.P. (2013). Parasympathetic stimulation improves epithelial organ regeneration. Nat. Commun..

[81] Emmerson E., May A.J., Nathan S., Cruz-Pacheco N., Lizama C.O., Maliskova L., Zovein A.C., Shen Y., Muench M.O., Knox S.M. (2017). SOX2 regulates acinar cell development in the salivary gland. Elife.

[82] Srinivasan R., Chang W.W. (1977). Effect of neonatal sympathectomy on the postnatal differentiation of the submandibular gland of the rat. Cell Tissue Res..

[83] Bloom G.D., Carlsoo B., Danielsson A., Hellstrom S., Henriksson R. (1981). Trophic effect of the sympathetic nervous system on the early development of the rat parotid gland: A quantitative ultrastructural study. Anat. Rec..

[84] Wheeler E.F., Bothwell M. (1992). Spatiotemporal patterns of expression of NGF and the low-affinity NGF receptor in rat embryos suggest functional roles in tissue morphogenesis and myogenesis. J. Neurosci..

[85] Borden P., Houtz J., Leach S.D., Kuruvilla R. (2013). Sympathetic innervation during development is necessary for pancreatic islet architecture and functional maturation. Cell Rep..

[86] Airaksinen M.S., Saarma M. (2002). The GDNF family: Signalling, biological functions and therapeutic value. Nat. Rev. Neurosci..

[87] Wang K., Demir I.E., D’Haese J.G., Tieftrunk E., Kujundzic K., Schorn S., Xing B., Kehl T., Friess H., Ceyhan G.O. (2014). The neurotrophic factor neurturin contributes toward an aggressive cancer cell phenotype, neuropathic pain and neuronal plasticity in pancreatic cancer. Carcinogenesis.

[88] Ceyhan G.O., Schafer K.H., Kerscher A.G., Rauch U., Demir I.E., Kadihasanoglu M., Bohm C., Muller M.W., Buchler M.W., Giese N.A. (2010). Nerve growth factor and artemin are paracrine mediators of pancreatic neuropathy in pancreatic adenocarcinoma. Ann. Surg..

[89] Pundavela J., Demont Y., Jobling P., Lincz L.F., Roselli S., Thorne R.F., Bond D., Bradshaw R.A., Walker M.M., Hondermarck H. (2014). ProNGF correlates with Gleason score and is a potential driver of nerve infiltration in prostate cancer. Am. J. Pathol..

[90] Dobrenis K., Gauthier L.R., Barroca V., Magnon C. (2015). Granulocyte colony-stimulating factor off-target effect on nerve outgrowth promotes prostate cancer development. Int. J. Cancer.

[91] Mauffrey P., Tchitchek N., Barroca V., Bemelmans A.P., Firlej V., Allory Y., Romeo P.H., Magnon C. (2019). Progenitors from the central nervous system drive neurogenesis in cancer. Nature.

[92] Madeo M., Colbert P.L., Vermeer D.W., Lucido C.T., Cain J.T., Vichaya E.G., Grossberg A.J., Muirhead D., Rickel A.P., Hong Z. (2018). Cancer exosomes induce tumor innervation. Nat. Commun..

[93] Lucido C.T., Wynja E., Madeo M., Williamson C.S., Schwartz L.E., Imblum B.A., Drapkin R., Vermeer P.D. (2019). Innervation of cervical carcinoma is mediated by cancer-derived exosomes. Gynecol. Oncol..

[94] Ching R.C., Wiberg M., Kingham P.J. (2018). Schwann cell-like differentiated adipose stem cells promote neurite outgrowth via secreted exosomes and RNA transfer. Stem Cell Res. Ther..

[95] Amit M., Takahashi H., Dragomir M.P., Lindemann A., Gleber-Netto F.O., Pickering C.R., Anfossi S., Osman A.A., Cai Y., Wang R. (2020). Loss of p53 drives neuron reprogramming in head and neck cancer. Nature.

[96] Marin-Acevedo J.A., Soyano A.E., Dholaria B., Knutson K.L., Lou Y. (2018). Cancer immunotherapy beyond immune checkpoint inhibitors. J. Hematol. Oncol..

[97] Weiss S.A., Wolchok J.D., Sznol M. (2019). Immunotherapy of melanoma: Facts and hopes. Clin. Cancer Res..

[98] Massarelli E., Papadimitrakopoulou V., Welsh J., Tang C., Tsao A.S. (2014). Immunotherapy in lung cancer. Transl. Lung Cancer Res..

[99] O’Donnell J.S., Teng M.W.L., Smyth M.J. (2019). Cancer immunoediting and resistance to T cell-based immunotherapy. Nat. Rev. Clin. Oncol..

[100] Restifo N.P., Antony P.A., Finkelstein S.E., Leitner W.W., Surman D.P., Theoret M.R., Touloukian C.E. (2002). Assumptions of the tumor ‘escape’ hypothesis. Semin. Cancer Biol..

[101] Beatty G.L., Gladney W.L. (2015). Immune escape mechanisms as a guide for cancer immunotherapy. Clin. Cancer Res..

[102] Larkin J., Chiarion-Sileni V., Gonzalez R., Grob J.J., Rutkowski P., Lao C.D., Cowey C.L., Schadendorf D., Wagstaff J., Dummer R. (2019). Five-Year survival with combined nivolumab and ipilimumab in advanced melanoma. N. Engl. J. Med..

[103] Conry R.M., Westbrook B., McKee S., Norwood T.G. (2018). Talimogene laherparepvec: First in class oncolytic virotherapy. Hum. Vaccin. Immunother..

[104] Anassi E., Ndefo U.A. (2011). Sipuleucel-T (provenge) injection: The first immunotherapy agent (vaccine) for hormone-refractory prostate cancer. Pharm. Ther..

[105] Pehlivan K.C., Duncan B.B., Lee D.W. (2018). CAR-T cell therapy for acute lymphoblastic leukemia: Transforming the treatment of relapsed and refractory disease. Curr. Hematol. Malig. Rep..

[106] Vaddepally R.K., Kharel P., Pandey R., Garje R., Chandra A.B. (2020). Review of indications of FDA-approved immune checkpoint inhibitors per NCCN guidelines with the level of evidence. Cancers.

[107] Muraille E., Leo O. (1998). Revisiting the Th1/Th2 paradigm. Scand. J. Immunol..

[108] Lenschow D.J., Walunas T.L., Bluestone J.A. (1996). CD28/B7 system of T cell costimulation. Annu. Rev. Immunol..

[109] Walunas T.L., Lenschow D.J., Bakker C.Y., Linsley P.S., Freeman G.J., Green J.M., Thompson C.B., Bluestone J.A. (1994). CTLA-4 can function as a negative regulator of T cell activation. Immunity.

[110] Agata Y., Kawasaki A., Nishimura H., Ishida Y., Tsubata T., Yagita H., Honjo T. (1996). Expression of the PD-1 antigen on the surface of stimulated mouse T and B lymphocytes. Int. Immunol..

[111] Kane L.P., Andres P.G., Howland K.C., Abbas A.K., Weiss A. (2001). Akt provides the CD28 costimulatory signal for up-regulation of IL-2 and IFN-gamma but not TH2 cytokines. Nat. Immunol..

[112] Pages F., Ragueneau M., Rottapel R., Truneh A., Nunes J., Imbert J., Olive D. (1994). Binding of phosphatidylinositol-3-OH kinase to CD28 is required for T-cell signalling. Nature.

[113] Keir M.E., Liang S.C., Guleria I., Latchman Y.E., Qipo A., Albacker L.A., Koulmanda M., Freeman G.J., Sayegh M.H., Sharpe A.H. (2006). Tissue expression of PD-L1 mediates peripheral T cell tolerance. J. Exp. Med..

[114] Robbins P.D., Morelli A.E. (2014). Regulation of immune responses by extracellular vesicles. Nat. Rev. Immunol..

[115] Maybruck B.T., Pfannenstiel L.W., Diaz-Montero M., Gastman B.R. (2017). Tumor-derived exosomes induce CD8(+) T cell suppressors. J. Immunother. Cancer.

[116] Bland C.L., Byrne-Hoffman C.N., Fernandez A., Rellick S.L., Deng W., Klinke D.J. (2018). Exosomes derived from B16F0 melanoma cells alter the transcriptome of cytotoxic T cells that impacts mitochondrial respiration. FEBS J..

[117] Olingy C.E., Dinh H.Q., Hedrick C.C. (2019). Monocyte heterogeneity and functions in cancer. J. Leukoc. Biol..

[118] Hanna R.N., Cekic C., Sag D., Tacke R., Thomas G.D., Nowyhed H., Herrley E., Rasquinha N., McArdle S., Wu R. (2015). Patrolling monocytes control tumor metastasis to the lung. Science.

[119] Hinshaw D.C., Shevde L.A. (2019). The tumor microenvironment innately modulates cancer progression. Cancer Res..

[120] Pathria P., Louis T.L., Varner J.A. (2019). Targeting tumor-associated macrophages in cancer. Trends Immunol..

[121] Zhao X., Qu J., Sun Y., Wang J., Liu X., Wang F., Zhang H., Wang W., Ma X., Gao X. (2017). Prognostic significance of tumor-associated macrophages in breast cancer: A meta-analysis of the literature. Oncotarget.

[122] Wang H.W., Joyce J.A. (2010). Alternative activation of tumor-associated macrophages by IL-4: Priming for protumoral functions. Cell Cycle.

[123] Zheng X., Turkowski K., Mora J., Brune B., Seeger W., Weigert A., Savai R. (2017). Redirecting tumor-associated macrophages to become tumoricidal effectors as a novel strategy for cancer therapy. Oncotarget.

[124] Plebanek M.P., Angeloni N.L., Vinokour E., Li J., Henkin A., Martinez-Marin D., Filleur S., Bhowmick R., Henkin J., Miller S.D. (2017). Pre-metastatic cancer exosomes induce immune surveillance by patrolling monocytes at the metastatic niche. Nat. Commun..

[125] Kanlikilicer P., Bayraktar R., Denizli M., Rashed M.H., Ivan C., Aslan B., Mitra R., Karagoz K., Bayraktar E., Zhang X. (2018). Exosomal miRNA confers chemo resistance via targeting Cav1/p-gp/M2-type macrophage axis in ovarian cancer. EBioMedicine.

[126] Cooks T., Pateras I.S., Jenkins L.M., Patel K.M., Robles A.I., Morris J., Forshew T., Appella E., Gorgoulis V.G., Harris C.C. (2018). Mutant p53 cancers reprogram macrophages to tumor supporting macrophages via exosomal miR-1246. Nat. Commun..

[127] Park J.E., Dutta B., Tse S.W., Gupta N., Tan C.F., Low J.K., Yeoh K.W., Kon O.L., Tam J.P., Sze S.K. (2019). Hypoxia-induced tumor exosomes promote M2-like macrophage polarization of infiltrating myeloid cells and microRNA-mediated metabolic shift. Oncogene.

[128] Binenbaum Y., Fridman E., Yaari Z., Milman N., Schroeder A., Ben David G., Shlomi T., Gil Z. (2018). Transfer of miRNA in macrophage-derived exosomes induces drug resistance in pancreatic adenocarcinoma. Cancer Res..

[129] Challagundla K.B., Wise P.M., Neviani P., Chava H., Murtadha M., Xu T., Kennedy R., Ivan C., Zhang X., Vannini I. (2015). Exosome-mediated transfer of microRNAs within the tumor microenvironment and neuroblastoma resistance to chemotherapy. J. Natl. Cancer Inst..

[130] Frank A.C., Ebersberger S., Fink A.F., Lampe S., Weigert A., Schmid T., Ebersberger I., Syed S.N., Brune B. (2019). Apoptotic tumor cell-derived microRNA-375 uses CD36 to alter the tumor-associated macrophage phenotype. Nat. Commun..

[131] Chen X., Ying X., Wang X., Wu X., Zhu Q., Wang X. (2017). Exosomes derived from hypoxic epithelial ovarian cancer deliver microRNA-940 to induce macrophage M2 polarization. Oncol. Rep..

[132] Gabrilovich D.I., Nagaraj S. (2009). Myeloid-derived suppressor cells as regulators of the immune system. Nat. Rev. Immunol..

[133] Condamine T., Ramachandran I., Youn J.I., Gabrilovich D.I. (2015). Regulation of tumor metastasis by myeloid-derived suppressor cells. Annu. Rev. Med..

[134] Guo X., Qiu W., Liu Q., Qian M., Wang S., Zhang Z., Gao X., Chen Z., Xue H., Li G. (2018). Immunosuppressive effects of hypoxia-induced glioma exosomes through myeloid-derived suppressor cells via the miR-10a/Rora and miR-21/Pten Pathways. Oncogene.

[135] Guo X., Qiu W., Wang J., Liu Q., Qian M., Wang S., Zhang Z., Gao X., Chen Z., Guo Q. (2019). Glioma exosomes mediate the expansion and function of myeloid-derived suppressor cells through microRNA-29a/Hbp1 and microRNA-92a/Prkar1a pathways. Int. J. Cancer.

[136] Langers I., Renoux V.M., Thiry M., Delvenne P., Jacobs N. (2012). Natural killer cells: Role in local tumor growth and metastasis. Biologics.

[137] Imai K., Matsuyama S., Miyake S., Suga K., Nakachi K. (2000). Natural cytotoxic activity of peripheral-blood lymphocytes and cancer incidence: An 11-year follow-up study of a general population. Lancet.

[138] Maurer S., Kropp K.N., Klein G., Steinle A., Haen S.P., Walz J.S., Hinterleitner C., Marklin M., Kopp H.G., Salih H.R. (2018). Platelet-mediated shedding of NKG2D ligands impairs NK cell immune-surveillance of tumor cells. Oncoimmunology.

[139] Neviani P., Wise P.M., Murtadha M., Liu C.W., Wu C.H., Jong A.Y., Seeger R.C., Fabbri M. (2019). Natural killer-derived exosomal miR-186 inhibits neuroblastoma growth and immune escape mechanisms. Cancer Res..

[140] Collin M., Bigley V. (2018). Human dendritic cell subsets: An update. Immunology.

[141] Chaput N., Conforti R., Viaud S., Spatz A., Zitvogel L. (2008). The Janus face of dendritic cells in cancer. Oncogene.

[142] Aspord C., Pedroza-Gonzalez A., Gallegos M., Tindle S., Burton E.C., Su D., Marches F., Banchereau J., Palucka A.K. (2007). Breast cancer instructs dendritic cells to prime interleukin 13-secreting CD4+ T cells that facilitate tumor development. J. Exp. Med..

[143] Zhou M., Chen J., Zhou L., Chen W., Ding G., Cao L. (2014). Pancreatic cancer derived exosomes regulate the expression of TLR4 in dendritic cells via miR-203. Cell Immunol..

[144] Zahalka A.H., Arnal-Estape A., Maryanovich M., Nakahara F., Cruz C.D., Finley L.W.S., Frenette P.S. (2017). Adrenergic nerves activate an angio-metabolic switch in prostate cancer. Science.

[145] Mo R.J., Han Z.D., Liang Y.K., Ye J.H., Wu S.L., Lin S.X., Zhang Y.Q., Song S.D., Jiang F.N., Zhong W.D. (2019). Expression of PD-L1 in tumor-associated nerves correlates with reduced CD8(+) tumor-associated lymphocytes and poor prognosis in prostate cancer. Int. J. Cancer.

[146] Cavel O., Shomron O., Shabtay A., Vital J., Trejo-Leider L., Weizman N., Krelin Y., Fong Y., Wong R.J., Amit M. (2012). Endoneurial macrophages induce perineural invasion of pancreatic cancer cells by secretion of GDNF and activation of RET tyrosine kinase receptor. Cancer Res..

[147] Yang M.W., Tao L.Y., Jiang Y.S., Yang J.Y., Huo Y.M., Liu D.J., Li J., Fu X.L., He R., Lin C. (2020). Perineural invasion reprograms the immune microenvironment through cholinergic signaling in pancreatic ductal adenocarcinoma. Cancer Res..

[148] Amit M., Na’ara S., Leider-Trejo L., Binenbaum Y., Kulish N., Fridman E., Shabtai-Orbach A., Wong R.J., Gil Z. (2017). Upregulation of RET induces perineurial invasion of pancreatic adenocarcinoma. Oncogene.

[149] Simeoli R., Montague K., Jones H.R., Castaldi L., Chambers D., Kelleher J.H., Vacca V., Pitcher T., Grist J., Al-Ahdal H. (2017). Exosomal cargo including microRNA regulates sensory neuron to macrophage communication after nerve trauma. Nat. Commun..

[150] Frick L.R., Arcos M.L., Rapanelli M., Zappia M.P., Brocco M., Mongini C., Genaro A.M., Cremaschi G.A. (2009). Chronic restraint stress impairs T-cell immunity and promotes tumor progression in mice. Stress.

[151] Estrada L.D., Agac D., Farrar J.D. (2016). Sympathetic neural signaling via the beta2-adrenergic receptor suppresses T-cell receptor-mediated human and mouse CD8(+) T-cell effector function. Eur. J. Immunol..

[152] Daher C., Vimeux L., Stoeva R., Peranzoni E., Bismuth G., Wieduwild E., Lucas B., Donnadieu E., Bercovici N., Trautmann A. (2019). Blockade of beta-adrenergic receptors improves CD8(+) T-cell priming and cancer vaccine efficacy. Cancer Immunol. Res..

[153] Mohammadpour H., MacDonald C.R., Qiao G., Chen M., Dong B., Hylander B.L., McCarthy P.L., Abrams S.I., Repasky E.A. (2019). beta2 adrenergic receptor-mediated signaling regulates the immunosuppressive potential of myeloid-derived suppressor cells. J. Clin. Investig..

[154] Araujo L.P., Maricato J.T., Guereschi M.G., Takenaka M.C., Nascimento V.M., de Melo F.M., Quintana F.J., Brum P.C., Basso A.S. (2019). The sympathetic nervous system mitigates CNS autoimmunity via beta2-adrenergic receptor signaling in immune cells. Cell Rep..

[155] Cheng Y., Tang X.Y., Li Y.X., Zhao D.D., Cao Q.H., Wu H.X., Yang H.B., Hao K., Yang Y. (2019). Depression-Induced Neuropeptide Y Secretion promotes prostate cancer growth by recruiting myeloid cells. Clin. Cancer Res..

[156] Chida Y., Hamer M., Wardle J., Steptoe A. (2008). Do stress-related psychosocial factors contribute to cancer incidence and survival?. Nat. Clin. Pract. Oncol..

[157] Dragomir M., Chen B., Fu X., Calin G.A. (2018). Key questions about the checkpoint blockade-are microRNAs an answer?. Cancer Biol. Med..

[158] Bapat A.A., Munoz R.M., Von Hoff D.D., Han H. (2016). Blocking nerve growth factor signaling reduces the neural invasion potential of pancreatic cancer cells. PLoS ONE.

[159] Kokolus K.M., Zhang Y., Sivik J.M., Schmeck C., Zhu J., Repasky E.A., Drabick J.J., Schell T.D. (2018). Beta blocker use correlates with better overall survival in metastatic melanoma patients and improves the efficacy of immunotherapies in mice. Oncoimmunology.

